# Trends in use of e-cigarette device types and heated tobacco products from 2016 to 2020 in England

**DOI:** 10.1038/s41598-021-92617-x

**Published:** 2021-06-24

**Authors:** Harry Tattan-Birch, Jamie Brown, Lion Shahab, Sarah E. Jackson

**Affiliations:** Department of Behavioural Science and Health, Institute of Epidemiology and Health Care, University College London, 1-19 Torrington Place, Fitzrovia, London, WC1E 7HB UK

**Keywords:** Human behaviour, Public health, Addiction

## Abstract

This study examined use trends of e-cigarette devices types, heated tobacco products (HTPs) and e-liquid nicotine concentrations in England from 2016 to 2020. Data were from a representative repeat cross-sectional survey of adults aged 16 or older. Bayesian logistic regression was used to estimate proportions and 95% credible intervals (CrIs). Of 75,355 participants, 5.3% (weighted = 5.5%) were currently using e-cigarettes or HTPs, with the majority (98.7%) using e-cigarettes. Among e-cigarette users, 53.7% (CrI 52.0–55.1%) used tank devices, 23.7% (22.4–25.1%) mods, 17.3% (16.1–18.4%) pods, and 5.4% (4.7–6.2%) disposables. Tanks were the most widely used device type throughout 2016–2020. Mods were second until 2020, when pods overtook them. Among all e-cigarette/HTP users, prevalence of HTP use remains rare (3.4% in 2016 versus 4.2% in 2020), whereas JUUL use has risen from 3.4% in 2018 to 11.8% in 2020. Across all years, nicotine concentrations of ≤ 6 mg/ml were most widely (41.0%; 39.4–42.4%) and ≥ 20 mg/ml least widely used (4.1%; 3.4–4.9%). Among e-cigarette/HTP users, ex-smokers were more likely than current smokers to use mod and tank e-cigarettes, but less likely to use pods, disposables, JUUL and HTPs. In conclusion, despite growing popularity of pods and HTPs worldwide, refillable tank e-cigarettes remain the most widely used device type in England.

## Introduction

Heated aerosolized nicotine delivery systems (HANDS) are handheld devices that heat either nicotine-infused liquid or tobacco sticks, producing an aerosol that can be inhaled. These include electronic cigarettes (“e-cigarettes”) and heated tobacco products (HTPs). Over the past decade, HANDS—principally e-cigarettes—have eclipsed nicotine replacement therapy as the most widely used aids for stopping smoking in England^1^. e-Cigarettes encompass a variety of different products, from bulky mod e-cigarettes to small cigarette shaped cigalikes. These can vary considerably in their potential to produce toxicants and carcinogens^2^, delivery of nicotine^3,4^, and effectiveness in helping people stop smoking combustible cigarettes^5–7^. It is therefore important to explore how the number of people using different device types and nicotine concentrations is changing, alongside a regulatory environment that may incentivise or discourage use of certain products. In this paper, we explore trends in the use of different e-cigarette device types and heated tobacco products in England, from 2016 to 2020.

### e-Cigarette device type

The most commonly used form of HANDS in England are e-cigarettes: hand-held electronic devices that heat a liquid, called an e-liquid, in order to produce an aerosol for inhalation. e-Liquid usually contains nicotine alongside propylene glycol, glycerol and flavourings. e-Cigarette use is widely recognised as less harmful to health than cigarette smoking, since users are exposed to much lower levels of toxicants and carcinogens^2,8,9^. However, public health bodies have differing attitudes towards the overall impact of e-cigarettes on public health, with some emphasising their potential use for smoking cessation while others highlighting risks to young non-smokers^10^. The UK has taken a policy approach that attempts to benefit from the use of e-cigarettes for smoking cessation, while minimising risks from youth use^11^. Evidence from randomised controlled trials^5,7,12^ and observational studies^1,13^ indicates that nicotine e-cigarettes can increase the likelihood that people will succeed in their attempts to stop smoking cigarettes. But their effectiveness for smoking cessation may depend on the specific device used.

Disposable cigarette shaped devices, also known as cigalikes, were the first type of e-cigarette to enter the market in England. Compared with newer devices, these deliver less nicotine^4^ and, as a result, may be less effective at helping people quit smoking^6^.

Tank e-cigarettes have a rechargeable battery and a tank that can be replenished with bottled e-liquid. They come in a variety of shapes, but most are the size of a fountain pen. These refillable tank devices tend to have a fixed power output, so the temperature to which e-liquid is heated remains relatively constant. They can deliver a similar amount of nicotine to cigarettes and satisfy cravings to smoke^14^. Two recent randomised controlled trials demonstrated the effectiveness of tank e-cigarettes for smoking cessation. The first found that they almost doubled the rate of successfully quitting smoking after 12 months when compared with nicotine replacement therapy^5^. The second found that, when used in conjunction with nicotine patches, tank e-cigarettes increased abstinence when compared to nicotine patches used alone or with placebo e-cigarettes^12^.

Modified or modular (“mod”) e-cigarettes are assembled by users from a variety of parts (e.g. atomizers and batteries). These are also refillable and rechargeable; however, they often have variable power output. This means that users can adjust the temperature to which their e-liquid is heated and, thus, the amount of vapour and nicotine they inhale. This can be problematic because hotter e-liquid makes the production of carcinogenic carbonyls, like formaldehyde, more likely^2^. However, most users find the aerosol produced at these hotter temperatures to be aversive, so are unlikely to vape with such high power settings^15^.

Pod devices are the most recent type of e-cigarette to enter the market in England. These are small, low powered, rechargeable e-cigarettes that use disposable cartridges (or “pods”) full of e-liquid. Because of their low power output, the nicotine concentration in pod e-liquid usually needs to be much higher than in mod devices to produce the same amount of nicotine per puff^16^. They produce less vapour and lower carbonyl yields than higher powered devices^17^. We will also look specifically into use of one brand of pod e-cigarettes: JUUL. JUUL, a manufacturer of sleek pod e-cigarettes, has received intense scrutiny because of the rapid growth in popularity of their devices in the US, especially among young people^18^. JUUL were among the first to use e-liquids that contain nicotine salts rather than freebase nicotine. This nicotine salts formulation has a pH that is more similar to the extravascular fluid in the lung, allowing users to vape much higher concentrations of nicotine without experiencing irritation to the throat, which may explain their popularity^3,19^. Other pod manufacturers followed suit, adding nicotine salts to e-liquid in their cartridges^20^. Laboratory results show that pods using nicotine salts reduce urges to smoke more rapidly than other types of e-cigarettes, which might make them more effective at helping people quit smoking^3,20^. JUUL launched in England in the summer of 2018^21^.

In this study, we will explore how the proportion of people in England using disposable, tank, mod, and pod (including JUUL specifically) devices has changed from 2016 to 2020.

### Nicotine concentration

The amount of nicotine that vapers receive from their e-cigarette per puff depends on the nicotine concentration of the e-liquid, features of the device, such as power output and wick material, and the duration and strength with which they puff. Experimental evidence shows that people self-titrate their nicotine consumption when vaping, such that those who use low nicotine concentration e-liquids tend to puff on their device more often and for longer in order to achieve their desired nicotine intake and, as a result, inhale a greater volume of aerosol^22^. Moreover, people who use variable power devices can raise the temperature of their device, which increases e-liquid consumption and formaldehyde production^22,23^. Therefore, it is important to track how the popularity of various nicotine concentrations is changing in England, within the context of EU TPD regulation that limits nicotine concentration in e-liquid to ≤ 20 mg/ml. This nicotine cap may also influence the popularity of different device types. Most notably, it could suppress use of pod e-cigarettes, like JUUL, that are sold at much higher concentrations (up to 59 mg/ml) in countries without such regulations.

### Heated tobacco products

Another form of HANDS with growing popularity globally are heated (or “heat-not-burn”) tobacco products^24^, such as IQOS by Philip Morris International. These are handheld devices that heat tobacco to a high enough temperature to produce a nicotine-infused aerosol, but thought to be too low to cause combustion^25^. Toxins produced by burning tobacco are the primary cause of harm from cigarettes, so avoiding combustion likely substantially lowers the risk of tobacco-related diseases^26^. Unlike e-cigarettes, heated tobacco products contain tobacco leaf. Their flavour may more closely resemble cigarette smoke^27^, which could make them more appealing to smokers trying to quit. Before their entrance into the UK market in late 2016, heated tobacco products had become very popular in Japan and South Korea, and made up 15.8% and 8.0% of each country’s respective tobacco sales in 2018^24^. Yet, at least initially, the use of heated tobacco products was rare in England^26^. Here, we will explore whether the prevalence of heated tobacco product use in England has grown since 2017.

### Frequency of use

Along with other factors such as reason for vaping^28–30^, the effectiveness of e-cigarettes for smoking cessation likely depends on how frequently smokers use their e-cigarette: people who use e-cigarettes daily have higher odds of subsequently quitting smoking when compared with less frequent users^31^. Therefore, we will explore how use of different devices and nicotine concentrations vary between daily and non-daily HANDS users.

### Differences by smoking status

Vapers who also smoke (54%) might use different types of e-cigarettes than those who have quit smoking (40%) or never smoked (6%)^32^. For instance, devices that do not help people quit smoking would be rarely used by ex-smokers, because smokers who use them would be unlikely to transition to sole e-cigarette use. In this study, we investigate whether HANDS users who also smoke use different e-cigarette device types, heated tobacco products and nicotine concentrations than those who are former or never smokers.

### Research aims

To summarise, we aim to measure annual trends from 2016 to 2020 in England in:

1. the proportion of HANDS users who use different types of e-cigarette devices (e.g. tank devices) or heated tobacco products, and
2. the proportion of e-cigarette users who use e-liquids of various nicotine concentrations.

We also aim to compare how use of these products differs between (1) daily and non-daily HANDS users, and (2) HANDS users who are smokers, ex-smokers and never smokers.

## Methods

### Design

Data came from the Smoking Toolkit Study (STS), a monthly repeated cross-sectional survey that provides detailed information on smoking behaviours and e-cigarette use in England. Using a combination of random location and quota sampling, it recruits approximately 1700 participants per month^33^. Comparisons with other national surveys and sales data show that the STS recruits a representative sample of the population in England^34,35^. Ethical approval was provided by the UCL Research Ethics Committee (0498/001). Participants gave informed consent to take part in the study. All participants were at least the age required (≥ 16 years) to give informed consent under UK Health Research Authority guidelines^36^. All methods were carried out in accordance with relevant regulations and guidelines.

### Study sample

Adults aged ≥ 16 years who reported that they were currently using e-cigarettes or heated tobacco products (HTPs). Data were included from July 2016, the month where detailed e-cigarette usage characteristics were first recorded, through February 2020. Questions about use of JUUL and heated tobacco products were added to the survey in July 2018 and December 2016, respectively.

### Measures

#### Type of e-cigarette or heated tobacco product

Participants were asked a series of questions about whether they currently use e-cigarettes, JUUL and/or heated tobacco products to cut down the amount they smoke, in situations when they are not allowed to smoke, to help them stop smoking, or for any other reason at all. Their responses were categorised as follows:

- *e-Cigarette user—*“Electronic cigarette”
- *Heated tobacco product user—*“heat-not-burn cigarette (e.g. iQOS with HEETS, heatsticks)”
- *JUUL user—*“JUUL”

e-Cigarette (non-JUUL) users were asked a follow-up question about the specific device(s) they used: “Which of the following do you mainly use…?” They could respond:

- *Disposable*—“A disposable e-cigarette or vaping device (non-rechargeable)”
- *Tank*—“An e-cigarette or vaping device with a tank that you refill with liquids (rechargeable)”
- *Mod*—“A modular system that you refill with liquids (you use your own combination of separate devices: batteries, atomizers, etc.)”
- *Pod*—“An e-cigarette or vaping device that uses replaceable pre-filled cartridges (rechargeable)”

#### Frequency of use

HANDS users were asked: “How many times per day on average do you use your nicotine replacement product or products?” Those who reported using their e-cigarette or heated tobacco product at least once a day were classified as daily users. All others were considered non-daily users.

#### Nicotine concentration

e-Cigarette users (non-JUUL) were asked: “Does the electronic cigarette or vaping device you mainly use contain nicotine?” They could respond “yes”, “no”, or “don’t know”.

Participants who reported using a non-JUUL e-cigarette with nicotine were asked: “What strength is the e-liquid that you mainly use in your electronic cigarette or vaping device?”. They could respond:

- “6 mg/ml (~ 0.6%) or less”
- “7 mg/ml (~ 0.7%) to 11 mg/ml (~ 1.1%)”
- “12 mg/ml (~ 1.2%) to 19 mg/ml (~ 1.9%)”
- “20 mg/ml (~ 2.0%) or more”
- “Don’t know”

#### Source of purchase

We also measured trends in the types of vaping retailer that were most widely used by e-cigarette users. Details of these analyses are available in the “Supplementary material”.

#### Smoking status

Participants were asked which of the following best applied to them.

- “I smoke cigarettes (including hand-rolled) every day”
- “I smoke cigarettes (including hand-rolled), but not every day”
- “I do not smoke cigarettes at all, but I do smoke tobacco of some kind (e.g. pipe, cigar or shisha)”
- “I have stopped smoking completely in the last year”
- “I stopped smoking completely more than a year ago”
- “I have never been a smoker (i.e. smoked for a year or more)”

Those who reported currently smoking cigarettes or tobacco of another kind (responses a–c) were considered smokers, and those who reported stopping smoking within the last year or more than a year ago (responses d, e) were considered ex-smokers. All others (response f) were considered never-smokers.

#### Socio-demographic characteristics

Age, gender, ethnicity (white, minority ethnic), and occupation-based social grade (C2DE includes manual routine, semi-routine, lower supervisory, and long-term unemployed; ABC1 includes managerial, professional and upper supervisory occupations^37^) were recorded.

### Analysis

#### Analytic strategy

The analysis was conducted in R and Stan^38,39^. The pre-registered analysis plan is available on the Open Science Framework (https://osf.io/57fvd/). Bayesian inference was used throughout, which allowed us to (1) report the relative plausibility of parameter values given the model and data and (2) include weakly informative priors, which regularise estimates and thus reduce the risk of overfitting^40^. Following a conservative approach^41^, priors were selected using prior predictive simulation (details available on https://osf.io/57fvd/). 95% credible intervals (95% CIs) represent highest posterior density intervals. We only analysed cases with complete information on all the variables that were included in each model. Survey weights were applied to calculate the overall prevalence of HANDS use among adults. All other analyses were unweighted, as they were calculated from a small subsample of the population (current HANDS users).

#### Sample characteristics

The proportion of people with different socio-demographic characteristics who used each device type were reported descriptively.

#### Device type

We estimated the total proportion of HANDS users who reported using each different device type. To explore how device usage changed from 2016 to 2020, we constructed logistic regression models with year of survey as an explanatory variable. From these models, we reported the proportion of HANDS users who used each device type in each year, alongside 95% CIs. We then stratified by frequency of use, to compare relative risk (RR) of use of each device type between daily and non-daily users, excluding participants who used combinations of device types or nicotine replacement therapy (NRT).

#### Nicotine concentration

We estimated the total proportion of e-cigarette users who reported using each of the different nicotine concentrations listed in the measures section. We again constructed logistic models with year of survey as an explanatory variable. Yearly estimates of the proportion of e-cigarette users who used each nicotine concentration were reported alongside 95% CrIs. We then stratified by frequency of use, to compare daily vs. non-daily use of each nicotine concentration. Finally, we presented the proportion of users of each device type who used each nicotine concentration of e-liquid, excluding participants who used combinations of device types.

#### Difference by smoking status

To test whether there were differences in device type or nicotine concentration use between smokers, ex-smokers and never smokers, we constructed a set of logistic regression models for each outcome including smoking status as an explanatory variable.

## Results

Of the 75,355 adults who responded to the Smoking Toolkit Study between August 2016 and February 2020, 3786 (unweighted = 5.29%, weighted = 5.53%; 95% CrI 5.45–5.62%) reported currently using HANDS, and this prevalence remained relatively stable from 2016 to 2020 (see Supplementary Fig. S2). Socio-demographic information for users of each device type are shown in Table 1.

**Table 1:** Sample characteristics by type of device used (*n* = 3786).

	Among adults (*n* = 75,355)	Among HANDS^a^ users (*n* = 3786)					
	Any HANDS	Disposable	Pod	Tank	Mod	JUUL^b^	HTP^c^
**Age (years)**							
16–17	32 (2.9%)	1 (3.1%)	6 (18.8%)	18 (56.2%)	5 (15.6%)	1 (16.7%)	0 (0.0%)
18–24	554 (5.6%)	29 (5.2%)	76 (13.7%)	282 (50.9%)	121 (21.8%)	30 (12.1%)	8 (1.6%)
25–34	817 (7.7%)	33 (4.0%)	94 (11.5%)	414 (50.7%)	225 (27.5%)	13 (3.4%)	14 (1.9%)
35–44	711 (6.8%)	40 (5.6%)	112 (15.8%)	343 (48.2%)	163 (22.9%)	4 (1.4%)	19 (3.1%)
45–54	760 (6.9%)	34 (4.5%)	122 (16.1%)	397 (52.2%)	152 (20.0%)	10 (3.1%)	14 (2.1%)
55–64	636 (5.5%)	30 (4.7%)	123 (19.3%)	309 (48.6%)	129 (20.3%)	9 (3.1%)	10 (1.8%)
65+	463 (2.3%)	34 (7.3%)	102 (22.0%)	203 (43.8%)	76 (16.4%)	20 (9.3%)	12 (2.9%)
**Gender**							
Men	2192 (5.8%)	116 (5.3%)	313 (14.3%)	1087 (49.6%)	510 (23.3%)	56 (5.7%)	37 (1.9%)
Women	1787 (4.7%)	85 (4.8%)	323 (18.1%)	880 (49.2%)	362 (20.3%)	34 (4.4%)	41 (2.6%)
**Ethnicity**							
White	3630 (5.7%)	177 (4.9%)	569 (15.7%)	1823 (50.2%)	807 (22.2%)	68 (4.3%)	63 (2.0%)
Minority ethnic	343 (3.2%)	24 (7.0%)	64 (18.7%)	145 (42.3%)	61 (17.8%)	22 (12.2%)	14 (4.6%)
**Social grade**							
ABC1	2044 (4.5%)	83 (4.1%)	337 (16.5%)	1008 (49.3%)	445 (21.8%)	66 (7.1%)	37 (2.1%)
C2DE	1942 (6.5%)	118 (6.1%)	299 (15.4%)	964 (49.6%)	427 (22.0%)	24 (2.9%)	41 (2.4%)

The percentage who used each different device type are shown in brackets. People could report that they used multiple products.

^a^Heated aerosolized nicotine delivery systems (HANDS) include e-cigarettes and heated tobacco products.

^b^The denominator used to calculate percentages in this column was the number of HANDS users surveyed from July 2018—the month in which JUUL use began being recorded—to February 2020 (*n* = 1760).

^c^The denominator used to calculate percentages in this column was the number of HANDS users surveyed from December 2016—the month in which heated tobacco products (HTPs) use began being recorded—to February 2020 (*n* = 3520).

### Device type

Overall, e-cigarettes were used by 98.7% (95% CrI 98.4–99.0%) of HANDS users and heated tobacco products by 2.2% (1.8–2.7%). Among e-cigarette users, 53.7% (52.0–55.1%) used tank devices, 23.7% (22.4–25.1%) used mods, 17.3% (16.1–18.4%) used pods, and 5.4% (4.7–6.2%) used disposables. JUUL e-cigarettes were used by 5.1% (4.1–6.1%) of HANDS users.

**Figure 1:**
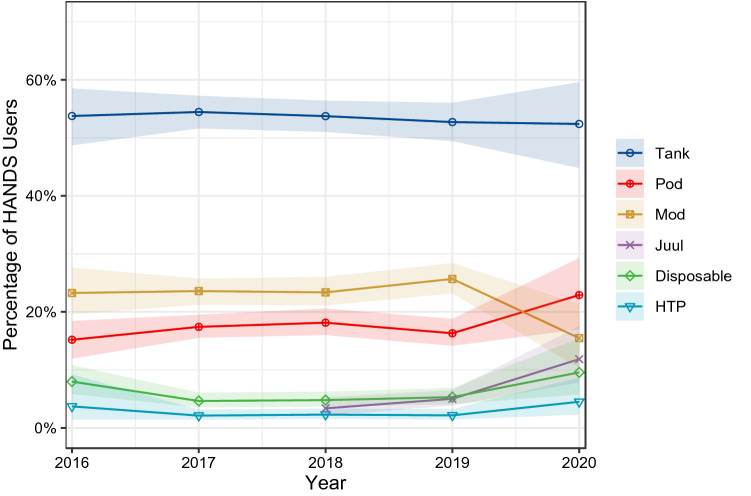
Use of different e-cigarette device types and heated tobacco product use among adult heated aerosolized nicotine delivery system (HANDS) users in England from 2016 to 2020. Shaded bands represent 95% CrIs. Trends would be the same with a denominator of all adults rather than only HANDS users, as overall prevalence of HANDS use was stable from 2016 to 2020 (Supplementary Fig. S2). Some people reported using JUUL but not pods. When these individuals were considered pod users, there was a more pronounced rise in pod use from 19.5% in 2019 to 30.4% in 2020.

Figure 1 shows trends in the prevalence of usage of different device types among HANDS users from 2016 to 2020. In general, use of different device types remained stable over time, with tank e-cigarette consistently the most widely used device type. Mod e-cigarettes were the second most commonly used device type until 2020, when pods overtook them. Use of JUUL has risen from 3.4% (2.1–5.3%) of HANDS users in 2018 to 11.8% (7.8–17.6%) in 2020. Heated tobacco product use has remained rare—with 3.4% (1.2–8.0%) of HANDS users using them in 2016 versus 4.2% (2.2–7.5%) in 2020. Relative to non-daily e-cigarette users, daily users were more likely to use tank devices, equally likely to use mods, but less likely to use disposables or pods (Table 2). HANDS users who currently smoked were less likely than those who never smoked to use JUUL and heated tobacco products, but more likely to use pods. Conversely, they were less likely to use mod and tank e-cigarettes than ex-smokers, but more likely to use pods, disposables, JUUL and heated tobacco products.

**Table 2:** Device type use by frequency of use and smoking status among people who reported using HANDS.

	Disposable (95% CrI)	Pod (95% CrI)	Tank (95% CrI)	Mod (95% CrI)	JUUL^a^ (95% CrICrI)	HTP^b^ (95% CrI)
**Frequency of use**^**c**^						
Non-daily (N = 938)						
%	6.0 (4.5–7.6)	21.5 (18.9–24.4)	48.9 (45.8–51.9)	23.3 (20.8–26.1)	–	–
RR^d^	Ref	Ref	Ref	Ref	–	–
Daily (N = 2337)						
%	4.1 (3.4–4.9)	14.3 (12.9–15.7)	57.4 (55.4–59.2)	24.1 (22.5–25.6)	–	–
RR	0.70 (0.48–0.92)	0.67 (0.57–0.78)	1.18 (1.09–1.25)	1.03 (0.91–1.17)	–	–
**Smoking status**						
Smoker (N = 2354)						
%	6.1 (5.1–7.3)	19.3 (17.7–20.9)	51.0 (48.9–53.0)	22.5 (21.0–24.3)	4.3 (3.2–5.8)	2.6 (2.0–3.3)
RR	Ref	Ref	Ref	Ref	Ref	Ref
Never smoker (N = 264)						
%	6.0 (3.5–10.2)	13.7 (9.8–18.8)	53.1 (47.3–59.0)	28.1 (22.5–34.6)	26.8^e^ (20.2–34.6)	4.9 (2.9–8.3)
RR	1.02 (0.52–1.65)	0.72 (0.50–0.97)	1.04 (0.92–1.16)	1.26 (0.99–1.57)	6.32 (3.89–8.82)	1.96 (0.95–3.05
Ex-smoker (N = 1364)						
%	2.6 (1.9–3.7)	14.3 (12.6–16.3)	58.1 (55.1–60.9)	25.0 (22.6–27.4)	1.6 (0.9–2.9)	1.1 (0.6–1.7)
RR	0.44 (0.28–0.61)	0.75 (0.63–0.86)	1.14 (1.06–1.21)	1.11 (0.98–1.26)	0.39 (0.16–0.67)	0.43 (0.21–0.65)

Heated aerosolized nicotine delivery systems (HANDS) include e-cigarettes and heated tobacco products. Percentages are unweighted.

^a^Use of JUUL was recorded from July 2018 (*n* = 1760). Frequency could not be assessed as all JUUL users had used combinations of products.

^b^Use of heated tobacco products (HTPs) was recorded from December 2016 (*n* = 3520). Frequency could not be assessed as all but one HTP user used combinations of products.

^c^Participants who used combinations of products or NRT were excluded.

^d^RR represent risk ratio.

^e^The high prevalence of JUUL use among never smoking HANDS users was primarily driven by data from a single month in a specific local authority area. Therefore, it likely represents a localised effect.

### Nicotine concentration

**Figure 2:**
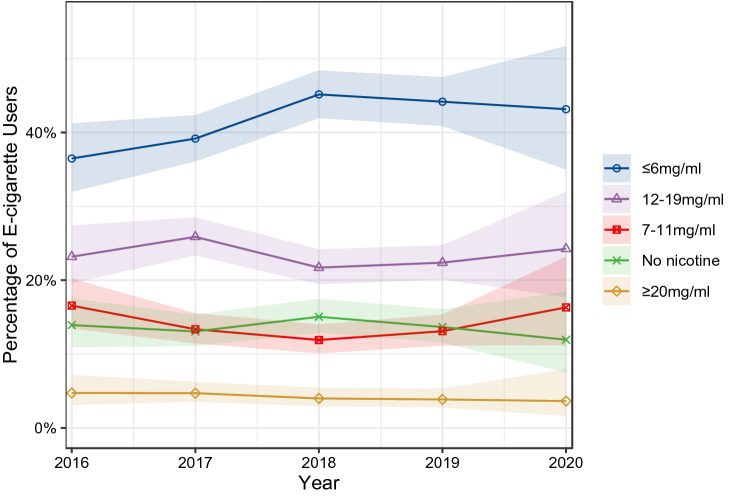
Nicotine concentration used in e-cigarettes by adult e-cigarette users in England from 2016 to 2020. Shaded bands represent 95% CrIs. Trends would be the same with a denominator of all adults rather than only e-cigarette users, as overall prevalence of e-cigarette use was stable from 2016 to 2020 (Supplementary Fig. S2).

The most widely used nicotine concentration was ≤ 6 mg/ml, used by 41.0% (39.4–42.4%) of e-cigarette users. This was followed by 12–19 mg/ml used by 23.4% (21.8–24.9%), 7–11 mg/ml by 13.4% (12.0–14.6%), no nicotine by 14.2% (13.2–15.1%), ≥ 20 mg/ml by 4.1% (3.4–4.9%), and do not know by 3.2% (2.6–3.8%). Figure 2 shows trends in use of different concentrations from 2016 to 2020. Use of different nicotine concentrations remained relatively stable, with ≤ 6 mg/ml being the most widely used concentration across all years. Relative to non-daily e-cigarette users, daily users were less likely to use non-nicotine e-liquid and more likely to use nicotine concentrations of ≤ 6 mg/ml and 12–19 mg/ml (Table 3). Use of non-nicotine e-liquid was more common among users of disposable e-cigarette than of other device types. Mod and tank users were more likely to use ≤ 6 mg/ml nicotine concentration than disposable e-cigarette users. Relative to never smokers, smokers and ex-smokers were less likely to use non-nicotine e-liquid and nicotine concentrations of ≥ 20 mg/ml.

**Table 3:** Nicotine concentration used by frequency of use, device type and smoking status among e-cigarette users.

	No nicotinic (95% CrI)	≤ 6 mg/ml (95% CrI)	7–11 mg/ml (95% CrI)	12–19 mg/ml (95% CrI)	≥ 20 mg/ml (95% CrI)	Do not know (95% CrI)
**Frequency of use**^**a**^						
Non-daily (N = 938)						
%	18.4 (16.3–20.5)	37.7 (35.0–40.6)	14.9 (13.1–16.9)	17.7 (15.7–19.8)	4.5 (3.5–5.9)	5.4 (4.3–6.7)
RR^**b**^	Ref	Ref	Ref	Ref	Ref	Ref
Daily (N = 2337)						
%	12.1 (11.0–13.4)	42.5 (40.6–44.2)	12.6 (11.4–14.0)	26.0 (24.4–27.6)	4.0 (3.3–4.8)	2.3 (1.8–3.0)
RR	0.66 (0.56–0.77)	1.13 (1.04–1.24)	0.85 (0.73–0.99)	1.47 (1.27–1.66)	0.89 (0.59–1.16)	0.44 (0.31–0.59)
**Device type**^**c**^						
Disposable (N = 153)						
%	21.9 (16.6–28.5)	31.3 (24.8–38.8)	16.4 (11.1–22.9)	20.4 (14.9–27.1)	1.4 (0.4–3.6)	7.4 (4.1–12.4)
RR	Ref	Ref	Ref	Ref	Ref	Ref
Pod (N = 538)						
%	15.2 (12.4–18.5)	30.7 (26.2–35.3)	16.0 (13.2–19.1)	26.3 (22.7–30.3)	5.8 (4.1–7.9)	5.0 (3.4–7.2)
RR	0.71 (0.49–0.96)	0.99 (0.73–1.24)	1.00 (0.58–1.40)	1.31 (0.90–1.78)	5.00 (1.28–11.69)	0.71 (0.32–1.20)
Tank (N = 1801)						
%	13.8 (12.3–15.4)	43.2 (41.1–45.6)	12.8 (11.4–14.4)	23.4 (21.8–25.3)	3.8 (3.0–4.8)	2.7 (2.0–3.5)
RR	0.64 (0.46–0.86)	1.39 (1.08–1.71)	0.80 (0.54–1.13)	1.17 (0.82–1.55)	3.27 (0.82–7.02)	0.38 (0.18–0.61)
Mod (N = 783)						
%	11.1 (8.9–13.5)	50.0 (46.9–53.2)	10.6 (8.2–13.1)	22.8 (19.9–26.1)	4.0 (2.9–5.4)	1.5 (0.8–2.6)
RR	0.52 (0.36–0.73)	1.61 (1.27–2.02)	0.67 (0.44–0.94)	1.14 (0.77–1.53)	3.45 (0.59–8.08)	0.23 (0.10–0.46)
**Smoking status**						
Smoker (N = 2266)						
%	14.5 (13.1–16.2)	39.9 (38.0–41.9)	15.2 (13.9–16.6)	21.0 (19.4–22.9)	3.8 (3.1–4.7)	4.4 (3.7–5.2)
RR	Ref	Ref	Ref	Ref	Ref	Ref
Never smoker (N = 250)						
%	23.4 (18.1–30.1)	40.3 (33.5–47.4)	9.3 (6.0–13.6)	18.0 (13.3–23.8)	7.0 (4.3–11.2)	2.8 (1.2–5.8)
RR	1.62 (1.17–2.02)	1.01 (0.83–1.18)	0.62 (0.40–0.90)	0.86 (0.61–1.10)	1.88 (1.04–2.87)	0.68 (0.19–1.23)
Ex-smoker (N = 1319)						
%	11.8 (10.2–13.7)	43.2 (40.7–45.8)	10.8 (9.4–12.5)	28.4 (26.4–30.8)	4.2 (3.3–5.3)	1.5 (0.9–2.4)
RR	0.82 (0.68–0.96)	1.08 (1.01–1.18)	0.72 (0.58–0.83)	1.35 (1.19–1.51)	1.10 (0.81–1.49)	0.36 (0.19–0.54)

^a^Percentages are unweighted.

^b^RR represents risk ratio.

^c^Participants who used combinations of products or NRT were excluded.

## Discussion

Tank e-cigarettes were consistently the most commonly used device type in England from 2016 to 2020 among adults who used HANDS. Mod e-cigarettes were the second most widely used device type until 2020, where pod e-cigarettes overtook them. JUUL use rose year-on-year from 2018 to the extent that JUUL is used by 1 in 10 people who use HANDS in 2020. Heated tobacco product use remains relatively rare (3.4% of HANDS users in 2016 versus 4.2% in 2020). Relative to non-daily e-cigarette users, daily users were more likely to use tank e-cigarettes but less likely to use disposables. Relative to HANDS users who were current smokers, those who were ex-smokers were more likely to use mod and tank e-cigarettes, but less likely to use pods, disposables, JUUL and heated tobacco products. In addition, prevalence of JUUL and heated tobacco product use was higher among HANDS users who were never smokers than ex- or current smokers. Across all years, the majority of e-cigarette users (> 80%) used e-liquid that contained nicotine, but lower nicotine concentrations (≤ 6 mg/ml) were most common. Daily e-cigarette users were less likely than non-daily users to use non-nicotine e-liquid. Relative to HANDS users who were ex- or current smokers, those who had never smoked were more likely to use both non-nicotine and high nicotine (≥ 20 mg/ml) e-liquid.

Comparison of these results with previous studies highlights differences between England and other countries^42–44^. Tank e-cigarettes have remained the most commonly used device type in England, while in the US pod e-cigarettes have become the most popular—primarily driven by the rise in JUUL use^18^. In the US, JUUL were widely and successfully marketed but advertising was much more limited in England by EU TPD regulations^18^. Pod e-cigarette and JUUL use rose modestly in England from 2019 to 2020. These differences could arise from the 20 mg/ml cap on nicotine concentration in e-cigarettes in the EU, which may have undermined how pod e-cigarettes with nicotine salts allow users to vape high nicotine concentrations without irritation to the throat^3,19^. However, an important recent product development may have changed this. JUUL have altered their products for the EU market such that each puff generates a greater volume of aerosol than equivalent products in America. Thus, products in both markets provide similar amounts of nicotine per puff despite using e-liquids with lower nicotine concentrations (18 mg/ml in EU versus 58 mg/ml in US)^45^. This change, enacted in summer 2019, may be one factor that has contributed to the recent increased prevalence of JUUL use in England. Further monitoring is required.

We found that use of heated tobacco products has remained relatively rare in England from 2016 to 2020, unlike in Japan and South Korea where these products have become increasingly popular^24,46,47^. This might result from England already having a well-established e-cigarette market when heated tobacco products launched in the country. Disposable cigarette use also remains rare in England, which is possibly a result of these products having poor nicotine delivery compared with other e-cigarettes^4^. Recently, a new type of disposable e-cigarette has entered markets across the world, which has a similar USB drive shape to pod devices^48^. These products use nicotine salts at similar concentrations to pod e-cigarettes like JUUL, and user reports suggest they may provide a stronger ‘hit’ than other disposable products^48^. This innovation may explain recent data showing an increase in use of disposable products among US youths^49^. Future research should track whether there are similar rises in the popularity of these disposable e-cigarettes in England.

As may be expected due to EU TPD regulation^50^, use of high nicotine concentrations (≥ 20 mg/ml) in England was rare. In fact, low (≤ 6 mg/ml) nicotine concentrations were most popular^51^. Although greater use of low nicotine concentrations may appear to benefit public health, the opposite may be the case. People tend to self-titrate their e-cigarette use to reach a desired nicotine level^22,23^. Thus, users of low nicotine concentration e-liquid may use their device more frequently, with longer puffs, and at hotter temperatures than those using high nicotine concentration e-liquids^52^, which could increase their risk of harm.

A sizeable minority of e-cigarette users (one-in-seven) reported using e-liquid without nicotine. However, as participants were asked for the nicotine concentration they “mainly use”, many of these individuals may have also used e-liquid with nicotine. This is especially important because shortfills—large bottles of non-nicotine e-liquids that are topped up with nicotine shots—are widely available in England. These shortfills allow users to circumvent the 10 ml bottle size cap for nicotine e-liquid introduced under EU TPD regulation^50^. Future research should measure whether participants use combinations of nicotine concentrations, to see the proportion of e-cigarette users that exclusively use e-liquid without nicotine.

We found differences in product use according to the frequency with which people vape. Relative to non-daily users, daily e-cigarette users were less likely to use disposable and pod devices and more likely to use tanks. This could indicate that people who try disposable e-cigarettes are unlikely to transition to more frequent use, possibly a result of them being less satisfying than other e-cigarettes^53^. Alternatively, frequent vapers may seek out products that are cheap to refill, like tanks, whereas those who vape infrequently may be more concerned about upfront cost of the e-cigarette device, preferring pods and disposables^54,55^. Non-daily e-cigarette users were more likely to use non-nicotine e-cigarettes than daily users. This is unsurprising given that nicotine is the primary dependence-inducing compound in e-cigarettes, which means use of non-nicotine products is unlikely to lead to dependence or more frequent use. It also indicates that, while 14% of e-cigarette users reported mainly using non-nicotine e-liquid, people used them less frequently, so the percentage share of the e-liquid market held by nicotine-free products is likely much lower than this.

There were also differences by smoking status. Pod, disposable, JUUL and HTP e-cigarette use was less common among HANDS users who were ex-smokers than current smokers. This might indicate that smokers who use these devices are less likely to quit smoking cigarettes. However, given the cross-sectional design of this study, it is difficult to infer how effective these products are for smoking cessation from these associations. An alternative explanation is that the convenience of pods and disposables relative to tanks and mods attracts people who want to use e-cigarettes to manage cravings to smoke rather than quit. Prevalence of JUUL and HTP use was higher among HANDS users who were never smokers than among those who were ex- or current smokers. However, this high prevalence was primarily driven by data from a single month in a specific local authority area, suggesting the difference may arise from a localised effect. Compared with current and ex-smokers, never smokers who used HANDS were more likely to use non-nicotine e-liquid. This is consistent with previous results showing minimal signs of nicotine dependence in e-cigarette users who have never smoked^56^. Prevalence of use of high (≥ 20 mg/ml) nicotine concentrations was low regardless of smoking status, but it was relatively higher among e-cigarettes users who were never smoker compared with those who were current or former smokers. However, there were very few never smokers in our sample, so this difference may be artefactual.

This study benefitted from using a representative sample of the population in England, having a pre-registered analysis plan, and measuring detailed information on participants’ usage of e-cigarettes, heated tobacco products and cigarettes. It also benefitted from the use of Bayesian 95% credible intervals, which possess the properties that researchers often misinterpret frequentist confidence intervals as having (i.e. there is a 95% probability that the true parameter value lies within the 95% credible interval, given the data and assumptions)^57,58^. However, there were several limitations. Firstly, participants who used combinations of HANDS device types and/or NRT (N = 377) were not asked about the nicotine concentration and frequency of use for each product separately, so they had to be excluded from some analyses. Secondly, there were less data for 2016 and 2020 than for other years, which meant that there was greater uncertainty around prevalence estimates. Results for these years may also differ from other years if product use varies across seasons, as estimates were calculated on data from only a few months in the year. In an unplanned sensitivity analysis, we found similar proportions of vapers using each product across all months, suggesting seasonality had little effect on results. Thirdly, there were very few participants surveyed among some subgroups (e.g. HANDS users who had never smoked), which meant there was large uncertainty around prevalence estimates.

## Conclusions

In England, choices of HANDS device types have remained relatively stable from 2016 to 2020, with tank e-cigarettes consistently the most widely used device type. Use of JUUL and heated tobacco products remains rare among HANDS users; however, there is some evidence JUUL use is becoming more common. Daily e-cigarette users were less likely to use disposable products. The vast majority of e-cigarette users used e-liquid that contained nicotine, but lower nicotine concentrations (≤ 6 mg/ml) were most popular. Relative to HANDS users who currently smoked, those who were ex-smokers were more likely to use mod and tank e-cigarettes, but less likely to use pods, disposables, JUUL and heated tobacco products. The e-cigarette and heated tobacco industries are adapting rapidly, with new innovations introduced each year. Regulations, such as nicotine caps, appear to shape which products people use. It is therefore essential that we continue to track the popularity of different devices and how it changes alongside a shifting regulatory landscape.

## Supplementary Information

Supplementary Information.

## Data Availability

Data are available upon reasonable request.
