# Supplementary material for: Trends in use of e-cigarette device types and heated tobacco products from 2016 to 2020 in England
Source: Sci Rep. 2021 Jun 24;11:13203. doi: 10.1038/s41598-021-92617-x (PMC8225841; doi:10.1038/s41598-021-92617-x)
Supplement: Supplementary file 1 — Supplementary Information. [file 41598_2021_92617_MOESM1_ESM.pdf]

Supplementary material

Trends in use of e-cigarette device types and heated tobacco products from 2016 to 2020 in England

Harry Tattan-Birch[1,2*], Jamie Brown[1,2], Lion Shahab[1,2], Sarah E. Jackson[1,2]

1. *Department of Behavioural Science and Health, University College London, London, UK*
2. *SPECTRUM Consortium, UK*

***Correspondence to:**

Harry Tattan-Birch

Email: h.tattan-birch@ucl.ac.uk | Telephone: +44 789 6878 330

Address: Institute of Epidemiology and Health Care, 1-19 Torrington Place, Fitzrovia, London WC1E 7HB

1. Source of Purchase

Introduction

Specialist vaping retailers have become commonplace in England, with dozens of new stores opening each quarter.[1] These tend not to sell tobacco cigarettes or cigars. Conversely, most newsagents, grocery stores and petrol stations now stock both cigarettes and vaping products.[2] In this study, we measure trends in the most common places people buy vaping products in England from 2016-2020. We might also expect that ex-smokers would prefer to purchase their vaping products from specialist stores rather than newsagents or supermarkets, which also stock tobacco cigarettes. Therefore, we examine differences in the source of purchase of vaping supplies between current smokers, ex-smokers and never smokers.

Research Aims

To summarise, we aim to measure annual trends from 2016 to 2020 in England in the proportion of e-cigarette users who purchase their vaping products from different types of retailer (e.g. supermarket, specialist vape store). We also aim to compare how source of purchase differ between e-cigarette users who are smokers, ex-smokers and never smokers.

Methods

Design

Data came from the Smoking Toolkit Study (STS), a monthly repeated cross-sectional survey that provides detailed information on smoking behaviours and e-cigarette use in England. Using a combination of random location and quota sampling, it recruits approximately 1700 participants per month.[3] Comparisons with other national surveys and sales data show that the STS recruits a representative sample of the population in England.[4,5]

Study Sample

Adults aged ≥16 years who reported that they were currently using e-cigarettes. Data were included from July 2016, the month where detailed e-cigarette usage characteristics were first recorded, through February 2020.

Measures

E-cigarette use

Participants were asked a series of questions about whether they currently use e-cigarettes to cut down the amount they smoke, in situations when they are not allowed to smoke, to help them stop smoking, or for any other reason at all. Those who said they currently used e-cigarettes for any reason were classified as "e-cigarette users".

Source of purchase

E-cigarette users (non-Juul) were asked: "From where do you usually buy your disposable e-cigarette or vaping device, pre-filled cartridges, e-liquids or electronic cigarette?" They could respond with one of the following types of retailer:

- *Vape shop* — "Specialist vape \ electronic cigarette retailer - not online"
- *Online vape retailer* — "Specialist vape \ electronic cigarette retailer – online"
- *Other online retailer* — "Other online retailer"
- *Newsagent* — "Newsagent\Off licence\Corner shop"
- *Petrol station* — "Petrol garage shop"
- *Supermarket* — "Supermarket"
- *Friend* — "Buy them cheap from friends"
- *Other* — "Other" or "Don't know"

Smoking status

Participants were asked which of the following best applied to them:

a) "I smoke cigarettes (including hand-rolled) every day"
b) "I smoke cigarettes (including hand-rolled), but not every day"
c) "I do not smoke cigarettes at all, but I do smoke tobacco of some kind (eg. pipe, cigar or shisha)"
d) "I have stopped smoking completely in the last year"
e) "I stopped smoking completely more than a year ago"
f) "I have never been a smoker (i.e. smoked for a year or more)"

Those who reported currently smoking cigarettes or tobacco of another kind (responses a-c) were considered smokers, and those who reported stopping smoking within the last year or more than a year ago (responses d-e) were considered ex-smokers. All others (response f) were considered never-smokers.

Socio-demographic characteristics

Age, gender, ethnicity (white, non-white), and occupation-based social grade (C2DE includes manual routine, semi-routine, lower supervisory, and long-term unemployed; ABC1 includes managerial, professional and upper supervisory occupations[6]) were recorded.

Analysis

Analytic strategy

The analysis was conducted in R and Stan.[7,8] Bayesian inference was used throughout, which allowed us to (i) report the relative plausibility of parameter values given the model and data and (ii) include weakly informative priors, which regularise estimates and thus reduce the risk of overfitting.[9] Following a conservative approach,[10] priors were selected using prior predictive simulation (see supplementary material). 95% credible intervals (95%CIs) represent highest posterior density intervals, which possess the properties that researchers often misinterpret frequentist confidence intervals as having.[11,12] We only included data from complete cases across variables included in each model.

We estimated the proportion of participants who reported purchasing their vaping products at each of the different types of retailers described in the measures section. We constructed a logistic regression model with year of survey as an explanatory variable. We report yearly estimates alongside 95%CIs.

To test whether there were differences in source of purchase between smokers, ex-smokers and never smokers, we constructed a set of logistic regression models for each outcome including smoking status as an explanatory variable.

Results

Overall, specialist vape shops were the most common source of purchase, with 42.1% (40.4%-43.6%) of e-cigarette users getting their vaping products from these stores. This was followed by supermarkets 16.8% (15.7%-18.0%), online vaping retailers 13.0% (12.0%-14.0%), newsagents 11.8% (10.9%-12.7%), other online retailers 6.8% (6.1%-7.9%), petrol stations 2.0% (1.6%-2.5%) and friends 1.8% (1.5%-2.4%). Supplementary Figure 1 shows the percentage of e-cigarette users who bought their products from each source from 2016 to 2020. Brick-and-mortar vape shops were consistently the most widely used retailer from 2016 to 2020. Supermarkets were the secondly most widely used retailer across all years, and they became increasingly popular from 2019 to 2020. Relative to e-cigarette users who were never smokers, ex-smokers were less likely to buy vaping products from friends (Supplementary Table 1). Moreover, ex-smokers and current smokers were less likely than never smokers to buy vaping products from non-specialist online retailers.

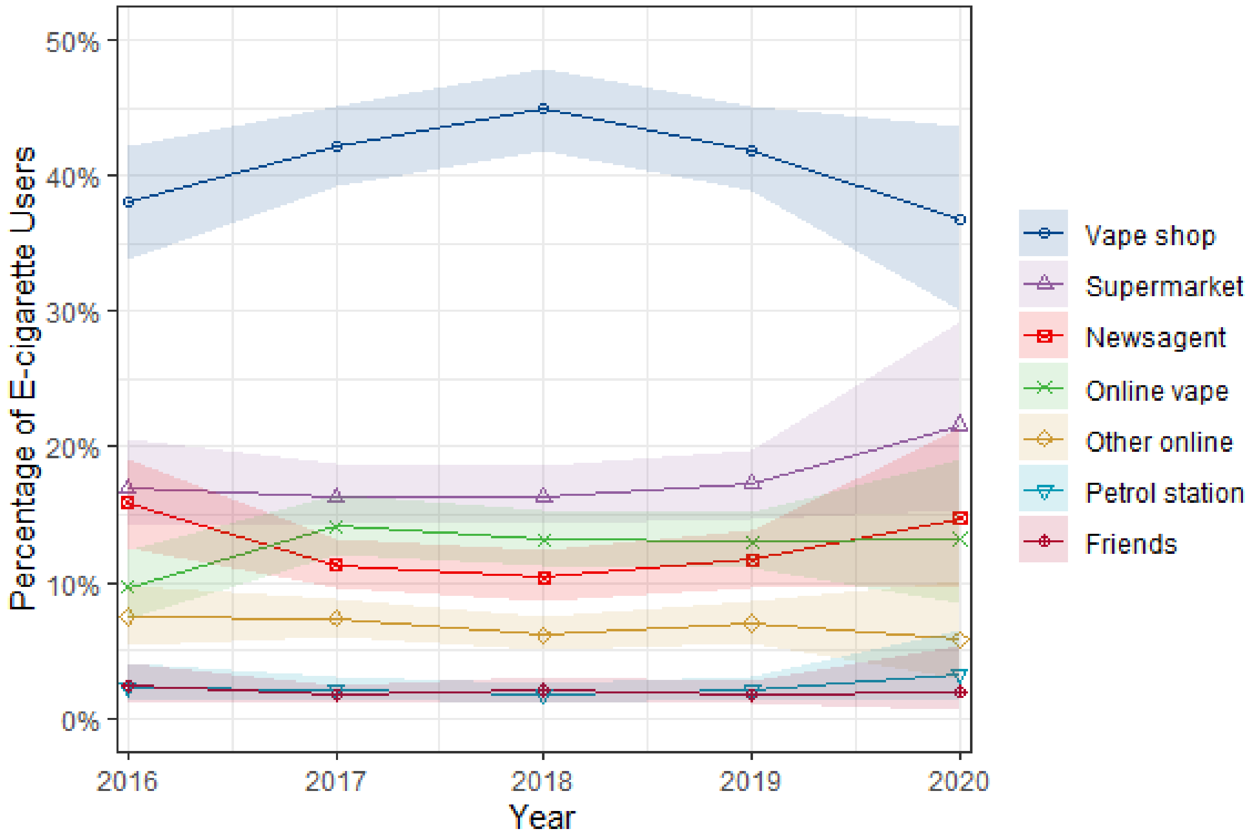

Supplementary Figure S1: Source of purchase of vaping supplies among adult e-cigarette users in England from 2016 to 2020. Shaded bands represent 95% CrIs.

Supplementary Table S1: Source of purchase of vaping supplies by smoking status among e-cigarette users.

		Vape shop (95% CrI)	Online vape retailer (95% CrI)	Other online retailer (95% CrI)	Newsagent (95% CrI)	Petrol station (95% CrI)	Supermarket (95% CrI)	Friends (95% CrI)	Other (95% CrI)
Never smoker	%	39.6 (33.2-45.7)	13.6 (9.8-19.0)	11.3 (7.9-16.0)	9.9 (6.7-14.5)	1.8 (0.6-4.1)	17.1 (12.4-22.5)	2.8 (1.1-5.6)	5.6 (3.2-9.1)
	RR	1.00	1.00	1.00	1.00	1.00	1.00	1.00	1.00
Ex-smoker	%	42.0 (39.4-44.8)	16.3 (14.3-18.5)	7.4 (6.2-9.0)	9.8 (8.3-11.3)	1.9 (1.2-2.6)	16.0 (13.9-18.0)	1.0 (0.6-1.6)	5.9 (4.7-7.2)
	RR	1.07 (0.88-1.27)	1.22 (0.85-1.68)	0.67 (0.44-0.94)	1.01 (0.61-1.43)	1.20 (0.36-2.73)	0.95 (0.65-1.24)	0.40 (0.12-0.86)	1.09 (0.55-1.71)
Smoker	%	42.5 (40.4-44.4)	10.9 (9.7-12.2)	6.0 (5.0-7.1)	13.2 (11.7-14.6)	2.1 (1.6-2.8)	17.4 (15.9-19.2)	2.3 (1.6-3.0)	5.8 (4.9-6.9)
	RR	1.08 (0.92-1.30)	0.82 (0.55-1.13)	0.54 (0.34-0.74)	1.36 (0.88-1.95)	1.34 (0.34-2.83)	1.03 (0.71-1.37)	0.89 (0.35-1.70)	1.08 (0.50-1.67)

Conclusions

From 2016 to 2020, around 40% of e-cigarette users mainly bought their vaping products from specialist, brick-and-mortar vape shops, making these the most widely used source of vaping products

in England. However, there seems to be a trend in from 2018 of supermarkets cutting into this share of the market, with increasing e-cigarette offers in stores. This is in contrast to the traditional tobacco retailers — newsagents and petrol stations — where less than 20% of e-cigarette users bought their vaping products. Never smokers were more likely than ex-smokers to buy vaping products from their friends. Nonetheless, absolute rates of purchase from friends remained low (<3%), regardless of smoking status.

References

1. PricewaterhouseCoopers UK. Store closures hit record levels. https://www.pwc.co.uk/press-room/press-releases/store-closures-hit-record-levels.html. Published 2019. Accessed March 6, 2020.
2. Braak D, Cummings K, Nahhas G, et al. Where Do Vapers Buy Their Vaping Supplies? Findings from the International Tobacco Control (ITC) 4 Country Smoking and Vaping Survey. *Int J Environ Res Public Health*. 2019;16(3):338. doi:10.3390/ijerph16030338
3. Fidler JA, Shahab L, West O, et al. “The smoking toolkit study”: a national study of smoking and smoking cessation in England. *BMC Public Health*. 2011;11(1):479. doi:10.1186/1471-2458-11-479
4. Fidler JA, Shahab L, West R. Strength of urges to smoke as a measure of severity of cigarette dependence: comparison with the Fagerström Test for Nicotine Dependence and its components. *Addiction*. 2011;106(3):631-638. doi:10.1111/j.1360-0443.2010.03226.x
5. Jackson SE, Beard E, Kujawski B, et al. Comparison of Trends in Self-reported Cigarette Consumption and Sales in England, 2011 to 2018. *JAMA Netw open*. 2019;2(8):e1910161. doi:10.1001/jamanetworkopen.2019.10161
6. Social Grade in National Readership Survey. http://www.nrs.co.uk/nrs-print/lifestyle-and-classification-data/social-grade/. Accessed November 7, 2019.
7. R Development Core Team . R: A Language and Environment for Statistical Computing. *R Found Stat Comput*. 2011. doi:10.1007/978-3-540-74686-7
8. Hoffman MD, Gelman A. *The No-U-Turn Sampler: Adaptively Setting Path Lengths in Hamiltonian Monte Carlo*. Vol 15.; 2014. http://mcmc-jags.sourceforge.net. Accessed December 22, 2019.
9. Lemoine NP. Moving beyond noninformative priors: why and how to choose weakly informative priors in Bayesian analyses. *Oikos*. 2019;128(7):912-928. doi:10.1111/oik.05985
10. McElreath R. *Statistical Rethinking : A Bayesian Course with Examples in R and Stan*.
11. Turkkan N, Pham-Gia T. Highest Posterior Density Credible Region and Minimum Area Confidence Region: the Bivariate Case. *J R Stat Soc Ser C (Applied Stat*. 1997;46(1):131-140.

doi:10.1111/1467-9876.00053

12. Hoekstra R, Morey RD, Rouder JN, Wagenmakers EJ. Robust misinterpretation of confidence intervals. *Psychon Bull Rev*. 2014;21(5):1157-1164. doi:10.3758/s13423-013-0572-3

2. Overall Trends

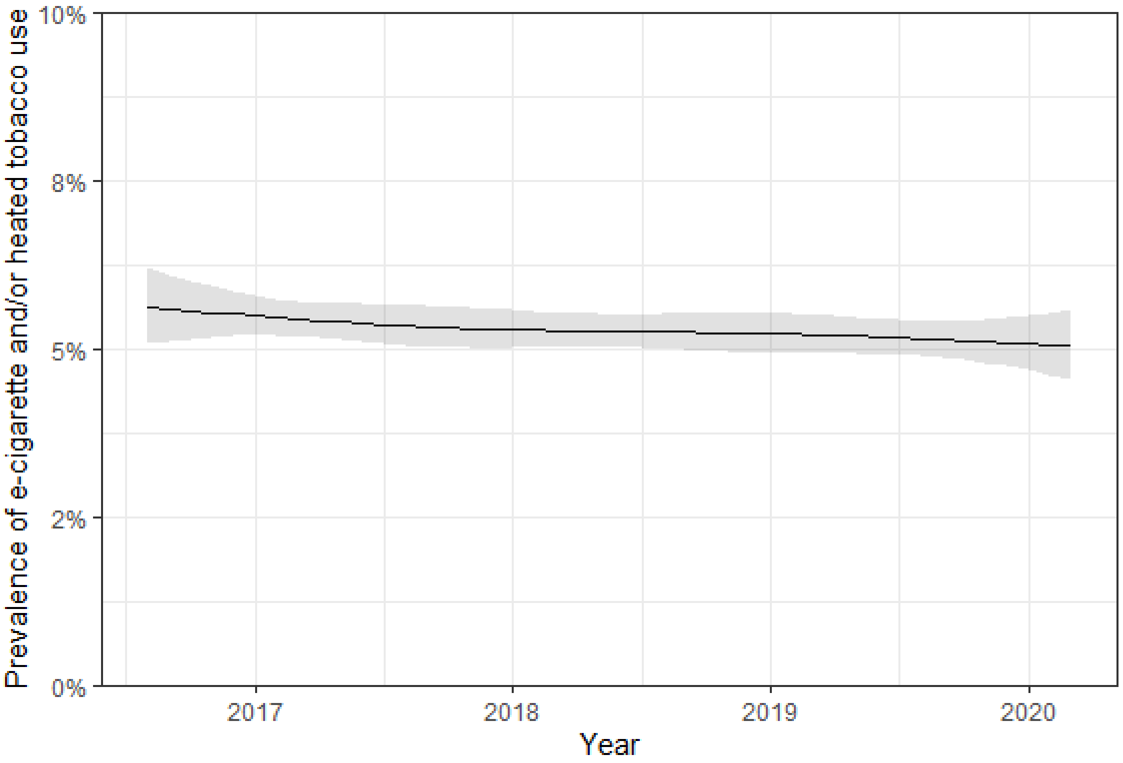

Supplementary Figure S2: Prevalence of e-cigarette and/or heated tobacco product use among adults (≥16) in England from 2016 to 2020. Shaded bands represent 95% CrIs. The trend is smoothed using natural cubic splines with four knots placed at quantiles of the data.
